# The lucky ones get cured: Health care seeking among women with pelvic organ prolapse in Amhara Region, Ethiopia

**DOI:** 10.1371/journal.pone.0207651

**Published:** 2018-11-26

**Authors:** Janne Lillelid Gjerde, Guri Rortveit, Mulat Adefris, Hibste Mekonnen, Tadesse Belayneh, Astrid Blystad

**Affiliations:** 1 Research group for Global Health Anthropology, Centre for International Health, Department of Global Public Health and Primary Care, University of Bergen, Bergen, Norway; 2 Department of Obstetrics and Gynecology, Haukeland University Hospital, Bergen, Norway; 3 Research Group for General Practice, Department of Global Public Health and Primary Care, University of Bergen, Bergen, Norway; 4 Research Unit for General Practice, Uni Research Health, Bergen, Norway; 5 Department of Obstetrics and Gynecology, College of Medicine and Health Sciences, University of Gondar, Gondar, Ethiopia; 6 Wogen Children and Mothers Support Association, Gondar, Ethiopia; 7 Department of Anaesthesiology, College of Medicine and Health Sciences, University of Gondar, Gondar, Ethiopia; University of Washington, UNITED STATES

## Abstract

**Background:**

The majority of women suffering from maternal morbidities live in resource-constrained settings with diverse barriers preventing access to quality biomedical health care services. This study aims to highlight the dynamics between the public health system and alternative healing through an exploration of the experiences of health care seeking among women living with severe symptomatic pelvic organ prolapse in an impoverished setting.

**Methods:**

The data were collected through ethnographic fieldwork at the hospital and community levels in the Amhara region of Ethiopia. The fieldwork included participant observation, 42 semi-structured interviews and two focus group discussions over a period of one year. A group of 24 women with severe symptomatic pelvic organ prolapse served as the study’s main informants. Other central groups of informants included health care providers, local healers and actors from the health authorities and non-governmental organisations.

**Results:**

Three case stories were chosen to illustrate the key findings related to health care seeking among the informants. The women strove to find remedies for their aggravating ailment, and many navigated between and combined various available healing options both within and beyond the health care sector. Their choices were strongly influenced by poverty, by lack of knowledge about the condition, by their religious and spiritual beliefs and by the shame and embarrassment related to the condition. An ongoing health campaign in the study area providing free surgical treatment for pelvic organ prolapse enabled a study of the experiences related to the introduction of free health services targeting maternal morbidity.

**Conclusions:**

This study highlights how structural barriers prevent women living in a resource-constrained setting from receiving health care for a highly prevalent and readily treatable maternal morbidity such as pelvic organ prolapse. Our results illustrate that the provision of free quality services may dramatically alter both health-and illness-related perceptions and conduct in an extremely vulnerable population.

## Introduction

Pelvic organ prolapse (hereafter ‘prolapse’) is the descent of one or more of the pelvic organs, and may be accompanied by symptoms such as bulging, heaviness and dragging sensations [1]. The condition is anatomically staged from 0 to IV as per the simplified Pelvic Organ Prolapse Quantification (S-POP) staging system [2], where stage IV implies a maximal descent and the entire extent of the vaginal mucosa everted. Studies have found that 6–7% of women in the US are affected by symptomatic prolapse [3, 4]. Pregnancy and childbirth are the most important risk factors [4, 5]. From resource constrained settings high parity, early pregnancy and heavy physical burdens are additional factors found to increase the risk of prolapse [6]. The condition can be treated through pelvic floor training exercises, by use of vaginal pessaries or by surgery. Vaginal pessaries are currently not commonly used in settings characterised by limited supply and health service availability [6]. Although surgical treatment for prolapse is available in urban areas in many low-income countries, large segments of the population, particularly those living in rural areas, have been found not to reach the health facilities due to distance, unaffordability and limited knowledge of the existing treatment combined with social stigma attached to the condition [7–10].

In Ethiopia the health care system consists of health posts, health centres, general/primary hospitals and referral hospitals [11]. Additionally there are various health facilities run by private actors, by non-governmental organisations (NGOs) and by mission- or faith-based actors [12]. In Ethiopia, 62% of government facilities charge routine user fees for general health services. Exemption schemes exist for particularly vulnerable groups, such as for obstetric or gynaecological emergencies. Additionally, many health facilities operate with a formal or informal system to waive fees for the very poorest [12]. A recent hospital-based study from North-West Ethiopia found that near 83% of women admitted with prolapse had delayed seeking help for an average of more than seven years, mainly due to unaffordability, [8], which indicates that the potential of receiving free treatment is not experienced as an easy option for this category of women. Their costs would under normal circumstances include payment for medicines and supplies during the hospital stay in addition to indirect costs for transport, lodging for accompanying family members and food [8]. Studies from Ethiopia moreover reveal that prolapse and other types of pelvic floor disorders are considered extremely sensitive, shameful and repulsive conditions, which further restricts women’s health seeking conduct [13–17]. Only 32% of women living in rural Ethiopia are literate, and many additionally live far from health facilities [11]. Living with a highly stigmatised condition in a context with severe lack of knowledge of how and where to get access to treatment in the public health system, will commonly lead women to search for healing options elsewhere [16].

In Ethiopia, it has been estimated that as many as 80% of the population search for care and cure outside of the public or private health facilities on a regular basis [18, 19]. Healing in Ethiopian ‘traditional’ medicine is commonly encompassing in its approach and is, according to Bishaw [20], concerned with the protection and promotion of human physical, spiritual, social, mental and material wellbeing. In the Amhara region, the most common means of healing are the use of local remedies, medicinal plants or spiritual guidance [18, 21] in addition to the common use of the religious ‘holy water’, or ‘*tsebel*’ in Amharic, which in the Ethiopian Orthodox Church is believed to facilitate spiritual and physical cure. The water is found in or around most Orthodox churches and usually originates from a natural spring thought to have been discovered by a saint or water blessed by a priest. The blessed water is commonly consumed in large amounts for days or weeks on end splashed on ailing body parts or showered in [21, 22].

In the present study we aimed to explore the experiences of health care seeking among women living with severe symptomatic prolapse in rural settings of the Amhara region in Ethiopia. We specifically aimed to explore what influences choices of health care and the dynamics between the public health services and other healing options. At the time of the research, a newly initiated health campaign was introduced in the study area through the regional governmental hospital. It was sponsored by international NGOs. The campaign involved trainings of health extension workers (community health workers) about prolapse conditions followed by community mobilisation and the recruitment and screening of women for prolapse in selected districts. The women who were willing to be assisted through surgery were provided with transport to the hospital and were provided free-of-charge surgical treatment for their prolapse. The campaign enabled us to explore potential transformations instigated by the introduction of free high-quality biomedical services in a context characterised by an impoverished rural population with minimal access to affordable health services. The discussion of the study findings will draw upon medical anthropological theory that emphasises structural dimensions more than cultural aspects in making sense of health seeking.

## Materials and methods

### Study design

A qualitative exploratory approach was employed in the current study. The data were collected through ethnographic fieldwork and implied participant observation, semi-structured qualitative interviews and focus group discussions.

### Study setting

The study took place at the University of Gondar Hospital (hereafter ‘the hospital’), a 500-bed comprehensive and specialised hospital located in the city of Gondar, and in semi-urban and rural communities within Dabat and Debark districts. All the study settings were located within the Amhara region in North-West Ethiopia.

Ethiopia has exceeded 100 million inhabitants and currently holds a total life expectancy of 65 years [23]. The nation’s fertility rate is 4.6 children per woman, and the percentage of institutional deliveries is 26% [11]. The maternal-mortality ratio remains high, with 412 deaths per 100,000 live births [11]. Despite documented improvements, the huge disparity between urban and rural residents remains evident. An indication is the fertility rate, estimated at 2.3 children per urban woman versus 5.2 children per rural woman, and that 80% of births to urban mothers are assisted by a skilled provider compared to 21% of births to rural mothers [11]. In the Amhara region people primarily practice Orthodox Christianity and speak Amharic as their first language [24]. The large majority of the rural population practice agriculture [11]. Around 54% of women and 42% of men have never attended school. The median female age upon first marriage in the region is 16 years, the lowest in the country [11].

### Study population and selection of participants

The 24 women who were enrolled at different stages of the newly initiated campaign and thus received free surgical treatment for their severe stages of prolapse served as the main informants in the present study (hereafter ‘the women’). Eight of the 24 women were followed up for a second interview in their homes some six to nine months following their surgery. The large majority of the women recruited to the study resided in the two study districts. The recruitment of the main informants stopped when no major new topics emerged, and thus followed the principles of data saturation. Other groups of supporting informants who had experience with the recruiting, referring or treating of women with prolapse were included in the study and added important contextual information (Table 1).

**Table 1:** Study participants according to recruitment place.

Hospital	n
Women admitted for free surgical treatment	19
Health workers	3
International NGO	1
**Community**	
Women with prolapse recently treated	4
Woman with prolapse awaiting treatment	1
Health extension workers	4
Health care providers at health centres	2
Local healers	4
International NGO	2
Health authorities	2
**Total number of informants**	**42**

### Data collection

The study was conducted over a period of 12 months in 2015–16 with repeated visits to the field. A total of eight months was spent in the study area by the first author (female), who is a Registered Nurse of background. Participant observation lasting for about three and a half months was carried out by the first author at the hospital and involved participating in nursing rounds, including assisting in the pre- and post-op care of patients, informal and systematic observation, communicating with health staff and stakeholders on the ward, interacting with the patients as well as identifying patients for interviews, and finally writing daily field notes. Follow-up interviews with some of the women who had undergone surgery involved numerous visits to the two study districts where the women lived, which increased the knowledge about the women’s home- and village-context through observations.

Healing through ‘*tsebel’* (holy water) was frequently brought up by the women even from the initial stages of the fieldwork. It was thus decided to include visits to holy water sites to learn more about what this common healing option implied. This visits to the locations of holy water involved participation in the morning prayer and/or the baptism in ‘*tsebel’* and interaction with women at the sites followed by the detailed writing of field notes. The use of local healers also emerged as important arenas for health care seeking among the women, and interviews with local healers were included in the study.

The interviews were carried out both at the hospital and in the communities by the first author in close collaboration with and the assistance of a local research assistant who was well acquainted with the culture, customs and language in the fieldwork area. The interviews normally lasted for one to two hours, and semi-structured interview guides with open-ended questions were employed with the aim to let the informant speak freely and with as few interruptions as possible. The interviews took place in a private room at the hospital or at the informant’s home or work place. The majority of the women had stayed days or weeks at the hospital at the time of the interview and had shared experiences among themselves as well as built up a certain level of trust in the researcher who interacted with them on a daily basis at the ward. During the interviews the majority of the women appeared somewhat shy but nonetheless willingly shared their experiences.

Towards the end of the fieldwork two focus group discussions were conducted at the hospital. These included a total of 12 women who were admitted to the hospital for free surgical treatment of prolapse through a later round of the campaign. Research assistants moderated the focus group discussions in Amharic and took notes. The participants were encouraged to speak freely and to each other within the overarching topic of health care seeking, and lasted for approximately one hour. An important aim of this method was to confirm seeming patterns and ambiguities in the already emergent findings of the study.

### Data analysis

The analysis took place throughout the data-collection process through discussions of emerging findings with the research assistant and more extensively between the field visits. After the completion of the fieldwork a more rigorous analytical phase guided by the writing of Miles and Huberman [25] was carried out. All interviews were audio-recorded, transcribed verbatim in Amharic and translated to English. The complete data material was carefully reviewed to identify core themes. The full dataset was imported into NVivo 11, a qualitative data-analysis software tool that was employed to organise the material and ease the analysis and data retrieval process. The main themes identified were organised into sub-categories followed by the coding of the material line by line. Each sub-category was scrutinised for central patterns, for ‘case-stories’ and for potential nuances, ambivalence and contradictions.

### Ethical considerations

Ethical approval was obtained from the Regional Committee for Medical and Health Research Ethics in Western-Norway on 25. August 2014 (2014/589) and from the Institutional Ethical Review Board of the University of Gondar, Ethiopia on 2. February 2015 (R/C/S/V/P/05/315/2015). The aim and purpose of the study as well as the contents of the consent form, including the assurance of anonymity, was read aloud to all informants prior to the interviews or focus group discussions. Written or oral consent to participate was obtained depending on literacy status. Oral consent was explicitly approved by the ethical committees for use among illiterate informants. Participants’ oral consent to participate in the study was recorded in writing for each study participant by the research assistant. Utmost care was taken to secure privacy and confidentiality during the interviews and throughout the entire research process. Approval was provided by the hospital to conduct participant observation and data collection at the relevant hospital ward. All patients on the ward were moreover provided with information about the study, the ongoing participant observation and their rights not to participate or to be observed. No patients declined to be observed; however, two women declined to be interviewed. In the two rural study districts, the heads of the district health administrations were informed about the purpose of the research project and were provided with an ethical approval letter. In the following presentation of the data, all names used are pseudonyms.

## Results

The 24 main informants had a mean age of 46 years (range 25–70). The majority of the women were married (17), while the remaining were either divorced (4) or widowed (3). Their mean age at first marriage was 13 years (range 7–19) and at first delivery near 18 years (range 13–24). They had an average of six children, and the majority (20) had delivered all their children at home, while the remaining (4) had had one or two deliveries at a health facility, often related to complications taking place during a home birth. All except one woman worked at home and were responsible for all household activities including cooking, cleaning, the fetching of water and firewood, taking care of children etc. Many (13) participated in the family’s agricultural activities. Of the 24 informants, 19 women had stage III prolapse, and 14 had lived with the prolapse for 10 years or longer. Five women had suffered from the condition for more than 20 years.

We will present three case stories, which serve as illustrations of typical choice scenarios women with prolapse are confronted with in this part of Ethiopia. The cases are based on the accounts of three of the interviewed women whose stories entail common healing trajectories emerging in the material. The stories highlight the women’s struggle to find solutions to their aggravating prolapse conditions within a complex landscape of healing options.

### Hanna’s story

We met Hanna at the hospital where she had recently been admitted via the ongoing prolapse surgery campaign. She was receiving treatment for severe ulceration of her prolapse prior to planned surgical treatment of her stage III prolapse. Hanna was a 45-year-old married woman from the rural lowland area, located far from accessible roads. As there was no access to a school in the lowland area where she resided, Hanna had never gone to school. She got married when she was 12 years old but strongly disliked her husband and managed to run away and get a divorce. Three years later she remarried and had nine children, seven of whom were still alive. She gave birth to all of her children at home. She explained to us that the prolapse had appeared around 20 years ago, after giving birth to her third child: ‘*I was shocked; it just came out spontaneously and ‘blocked me’ while I was in the middle of carrying out normal chores*. *Through time I got more used to it*’, she explained. Hanna was convinced that the prolapse was caused by her deliveries as well as by the hard work she had been engaged in since an early age: ‘*I was weeding*, *grinding*, *harvesting*, *fetching water and collecting fire wood*. *Back then we even used a stone mill*. *Women don’t get a rest; we are working all the time*. *Now at least we have electric mills*’. Despite the constraints experienced by the prolapse, she continued to strive to meet the expectations of a woman, wife and mother. She described her daily struggle saying: ‘*When I walked I used to feel like something pushed me down*, *like I had a cramp in my waist*. *I had to sit down and take periods of rest until it [the prolapse] went back inside and I could walk again*. *That’s how I used to live’*. After living with the condition for almost 10 years, Hanna decided to tell her older sister about her condition and ask for advice. She knew that she had suffered from a similar condition some years earlier. Her sister told her that she had herself received treatment for her prolapse at a hospital in a nearby city and got cured. Unlike her sister, who was much wealthier than her, Hanna realised that she could not afford the travel to the hospital, let alone the rest of the expenses related to the stay. ‘*When I thought about it*, *all I could think about was the expenses and the long travels*, *so I continued living my life*’. She did not consider disclosing to any of her friends or neighbours as she was afraid of being talked badly about or harassed if she spoke openly about her problem. However, 11 years ago when she was no longer able to hide the prolapse from her husband she decided to inform him. He did not say much but proved to be supportive as he shortly afterwards brought her to the hospital in Gondar to seek treatment. When they arrived all the beds were occupied, and she was told to wait. ‘*After I had waited for a month I lost hope*, *and we were about to return home*. *We had spent all our money*’. But just before returning home she was seen by a physician: ‘*He gave me a pill and an injection*. *That helped to ease my waist pain and dragging sensation*’. Back home, her prolapse soon started to prevent her from having intercourse, and as a result her husband initiated a relationship with another woman. When the pills that had proved helpful ran out, she decided to see a local healer. ‘*He asked me to go back [to the hospital]*, *but how could I*? *I asked him to give me more of those pills instead*, *and he did*. *It helped me for years’*. A year ago Hanna felt that the condition had worsened: ‘*Starting from harvesting time*, *it [the prolapse] went out completely*, *and it got an ulcer*. *I was thinking about going for treatment [at the hospital] but I still didn’t have the money’*. About a year later when Hanna heard the health extension workers inform about the possibility of free treatment of the condition she suffered from, she didn’t hesitate to approach them and was recruited for free treatment shortly afterwards.

#### Seeking help at health facilities

In our study, more than half (15 of 24) of the women had attempted to seek help from the public health facilities. However, the majority had only reached the local health centre in their district and never made it to the hospital where surgery could be carried out. A health officer at one of the health centres explained that the majority of the women they had referred to the hospital with severe stages of prolapse had returned back home due to lack of financial capacity after consultation at the health centre. One woman typically explained, ‘*I once went to the health centre*, *and they told me that I should go to the hospital to be operated on*. *But I had no money*. *Even if I wanted to go there*, *I couldn’t because I was broke’* (55-year-old divorced woman, treated for prolapse stage III). Some of the women moreover said that it was difficult to leave the household chores for a longer period of time. Others explained to us that a common negative perception of hospital treatment prevented them from going: ‘*I didn’t come [to the hospital] because I was scared of surgery*. *In our tradition surgery is perceived as death’* (Respondent 1, focus group discussion 1). The women who had previously managed to reach the hospital (five in total) had, similar to Hanna, experienced having to wait for a long time for the examination, reducing the limited funds available. Some had been sent home with remedies for ulceration treatment and been told to return once the wound had healed. Most had not managed to return. Besides the barriers of cost and time, the unsuccessful healing of the ulcer with the remedies they had been given made some of the women feel hesitant to return. The only woman who eventually returned did so through the strong support of her husband and by borrowing money from neighbours.

Unlike Hanna, the majority of our informants had not heard of others suffering from the same condition until the campaign. For years the women had thus lived with the condition not knowing what they were suffering from nor from where to seek help. According to a midwife working at one of the local health centres, hardly any women with prolapse sought help at the health centre on their own initiative: ‘*They say that they come because they are taught and sent by the health extension workers*. *No one came by themselves because they didn't know that their problem could be treated [at the hospital]’*. After being informed, examined and recruited for surgical treatment at the hospital, the women expressed relief and appreciation that the treatment was free, although for some the fear of surgery still remained: ‘*I’m half-hearted about the surgery*. *Part of me is worried*, *but on the other hand a part of me is telling me that it is better to have the surgery than continue living this way’* (40-year-old married woman, prolapse stage III).

### Aberash’s story

When we first met Aberash, a 50-year-old married woman, she had been admitted to the hospital. She got married at around 18 years old and had given birth to a total of seven children but lost two of them after having reached the health clinic. Aberash never went to school. She could not tell us exactly how long she had had the prolapse but explained that over the years she had gotten used to it and somehow managed to live with it. Two years ago, however, the prolapse suddenly increased in size and ‘*popped out*’: ‘*It reached a point where I couldn’t take it anymore*. *I didn’t know what my problem was*, *and I couldn’t tell or ask anyone about it*. *But I finally told some people’*. She decided to disclose to her close neighbours: ‘*They told me it was because the spirits are mad at me*. *Even my sisters said the same; they said “who else*?*”‘*. Accepting their explanation, Aberash sent her daughter to a local healer’s (‘*awaki’*) house to get instructions on how to respond to the spirits: ‘*I was told that I should conduct a ceremony three times together with my neighbours at home in order to heal*. *So I baked maize bread and prepared a meal from wheat flour and butter and brewed an alcoholic beverage called “araki”*. *I moreover slaughtered a red chicken and put on different clothes*, *a dress from homemade cotton’*. Her daughter, who recently had got a paid job, assisted her financially. However, the ceremonies did not improve Aberash’s condition: *‘They said the spirits will have mercy on me*, *but I was still sick after the three ceremonies’*. Although Aberash had been to the health clinic on several occasions with an injured eye and for a problem with her leg, she had never considered going there to seek help for the prolapse: *‘I didn’t know what was happening to me*. *I thought I was the only one this happened to*. *I was afraid to go to a hospital at this age for such a problem*, *so I thought it was better to stay at home’*. A year after conducting the ceremonies, at a Sunday mass in the church, she heard a woman talk about her recent experience of being treated for prolapse. The woman was talking in front of the crowd together with the health extension worker. Aberash decided to approach them. After being examined and found to have stage III prolapse with severe ulceration, she was recruited for free treatment. When we met Aberash, she had recently gone through the surgery and had done so with no doubts in her mind: ‘*I had already made my decision*. *I want to be normal*. *I didn’t have any fear’*.

#### Seeking cure through local healing options

Ten of the 25 women in our study informed that they had visited a healer to seek remedy due to the suffering related to their prolapse. Often they approached an ‘*awaki*’, ‘*tenquay*’ or ‘*yebahil hakim’*. These healers lived and operated in their local communities and offered diverse treatment options, also for prolapse. Most of the women had been asked by the healers to arrange ceremonies at their homes, like the ones conducted by Aberash. One woman had moreover been provided with small wooden sticks to be tied around her waist like a belt, while another had received a type of plant named ‘*kutegna*’ to be squeezed and inserted into the vagina. One woman who blamed her prolapse on being hit by the ‘evil eye’ had for years protected herself by staying inside the home as much as possible and by carrying an amulet made of animal skin containing a written script for protection (‘*yebuda medehanit’* or *‘kitab’*). Through visits to various ‘*tsebel*’ sites we observed women attempting to be healed from possession of what people claimed to be ‘evil eye’ through prayers, blessing with the cross and baptism in the holy water.

The four local healers interviewed in the present study all explained that they healed sickness through their ability to communicate with spirits such as ‘*qole*’ and ‘*wuqabe*’. One of the female healers known as an ‘*awaki*’ explained: *‘I have a “zaar” in my head [the opportunity to be possessed by the spirits]*. *I am sitting here as an obligation from the spirit [“zaar”]*. *I understand people’s problem as if they are my own’*. She had a clear opinion about the shortcomings of the treatment offered at the health facilities: *‘If a person has a problem with the spirits*, *and goes to the modern treatment*, *no doctor [health care provider] can treat her*. *The doctor wouldn’t understand the attack by the spirit*. *He wouldn’t know that she passed the limit of the spirits and that if she doesn’t provide something for the ‘qole’ [a type of spirit]*, *she might not get her health back’*. All the healers had frequently treated women suffering from urine leakage, but only one (an elderly ‘*awaki*’) said he had experience with the treatment of women with prolapse: ‘*I usually beg the ‘qole’ [a spirit]*, *and then their uterus goes back inside*. *Women who had the condition and who got treated came back and told me*. *I also asked them to beg [the spirits] by providing chickpea and sorghum and wheat bread in their home’*, he explained.

None of the women we talked to had experienced any long-term improvement from the treatment received by the local healers despite their belief in their potential to cure. The payments for the local healers’ services varied extensively–from no payment or other compensation until the treatment proved successful to extensive demands from the first consultancy. Several of our informants perceived treatment at healers as costly and demanding: ‘*We are asked for clothes*, *for a white goat*, *brown sheep or a red chicken*. *The “awakis” tell us that fulfilling these offers will heal us*, *but it’s only those who can afford it who can do that and get treated*. *It’s only those who are lucky who get any cure*. *How could a poor woman like me afford to buy a sheep*?*’* (Respondent 2, focus group discussion 2).

### Rahel’s story

We first met Rahel at the hospital. She was 39 years old, divorced and had suffered from prolapse for the previous 15 years. Rahel, like Hanna and Aberash, had never got the chance to go to school and was illiterate. To make a living she was a daily labourer, working as a cleaner and cook in wealthier people’s homes. She married the first time when she was nine years old. During the following years she experienced urinary leakage at night. Due to her problem, her husband started to disrespect her, and she decided to divorce him. She later remarried and had two children. Her second husband also disrespected and insulted her due to an aggravating condition of prolapse, and after six years together she decided to leave him and raise the children by herself. After her first divorce Rahel moved to the city and managed to save up money to seek help. She went to what she referred to as a ‘private clinic’ in the city, and although the person examining her admitted that he had never seen a condition like hers before, he provided her with some pills, an injection and ointment. This improved her situation for some time. However, the medicine was expensive and she never considered returning to him. She then moved back to the rural area, where she looked for other options for cure: ‘*There are a lot of ‘tsebel’ [holy water] places in our village*. *I tried at so many locations*. *They baptise and pray for the women who are seeking help*. *Once I stayed there for 15 days*, *another time for seven days*. *Whenever I got money I went and got baptised’*. She decided to disclose to the priests at the *‘tsebel’* site and felt well taken care of: ‘*I told them that I’m having a stomach-ache; that I feel pain in my stomach and that my uterus is coming out [prolapse–“mahatsene yiwetal” in Amharic]*. *Then they said that everything is going to be fine*, *and they baptised me in “tsebel” and blessed me with the cross*. *Next to God they were treating and supporting me a lot’*. While spending time at the *‘tsebel’* sites, Rahel realised that there were many other women suffering from the same condition as hers: *‘since we take off all of our clothes when we baptise we women can see each other’*. Still, she experienced openness around the condition as a challenge: ‘*Most of them did not talk about it unless we saw their body*, *and they covered it when we saw them*. *This problem is not easy to talk about openly*. *Some might even pretend they have another condition’*. Despite repeated visits to the *‘tsebel’* sites, Rahel did not experience any improvement of her condition. *‘I didn't get any hope [improvement] from the ‘tsebel’*, *but I didn't complain a lot*. *I thanked God for enabling me to work and feed myself*, *but I felt badly for not being able to socialise freely with other people’*. One day she was visiting a good friend of hers who had returned from the hospital after being ill: ‘*I went to her to ask her about how she is doing*. *We are very close*, *so I asked her about her sickness and she told me that her uterus was coming out [prolapse]*. *Then I asked her what they had done for her to improve her health*, *and I told her about my experience at the private clinic*. *She told me not to worry and that there is a new service being provided freely at the hospital’*. Rahel quickly afterwards consulted the health clinic, was examined and signed up for the ongoing campaign.

#### Seeking healing through holy water

Of the 24 women with prolapse in the current study, 18 explained that they had sought healing for their prolapse through the use of holy water. Many explained that they had been advised or influenced to do so by family members: ‘*I told my mother about my problem [the prolapse]*. *She was shocked and brought me straight to the holy water site’*, a 35-year-old woman explained. The women would typically stay at or visit the ‘*tsebel*’ site for 7, 14 or 21 days in order to complete a given treatment regime that included rigorous fasting, daily baptism, prayers and consumption of large amounts of ‘*tsebel*’. They would bring large jerry cans, which were filled with the blessed water during the time spent there. The cans were also by some brought back home for further consumption or baptism of their ailing bodies. The water was found in or around most Orthodox churches in their communities, and baptism in or consumption of the water normally implied a small fee that seemed to be affordable by our informants.

Unlike Rahel, the majority of our informants felt too ashamed and afraid to disclose the condition they suffered from to the priests or to other people seeking cure at the *‘tsebel’* site. Many attempted to hide the prolapse by covering it with their hand or by using underwear while baptising, although that was not unproblematic for all: *‘I tried to get baptised with my underwear on but it was stressful*. *I was trying to hide my problem from the other women*. *I was baptised once but it was discomforting*. *I didn’t go back after that because I was ashamed and afraid that people would find out about it [the prolapse]’* (70-year-old married woman, not yet diagnosed).

Repeated visits to the *‘tsebel’* sites without experiencing the expected or hoped for improvement of the condition had left many, like Rahel, frustrated and in despair. For the ones who had disclosed to others at the *‘tsebel’* site, some experienced support in their search for a solution: ‘*Then [after disclosing to each other] we started talking about it*. *After hearing about the free treatment being provided*, *we discussed and decided that the holy water couldn’t give us any solution regarding our problem*. *The only option was to get surgery*. *I went to the health worker and told them about my problem*. *We [she and the other woman with prolapse] came here together and now two got the surgery’* (35-year-old married woman, treated for prolapse stage III).

### Navigation between available health care options

No clear pattern emerged as to what and in which order of priority the women sought help for their condition. The conversation among the women in the focus group discussions illustrated further the diversity within health care seeking in the area: ‘*Firstly*, *people go to “awaki bet” [local healer’s house]*. *Then if there is no improvement people go to the holy water site and to the health centre*. *It is said that we need to beg the spirits first if we want to succeed*. *And then later if it gets worse we go to the holy water site or to the health facilities’*, one participant explained. A second participant argued for a different common pattern: ‘*People go to the health facility when they get money in hand*. *Then people say*: *“the doctors made it better; now it’s time for ‘tsebel’ [holy water]”‘*. A third participant moreover claimed that gender differences played a role in women’s health care seeking: *‘If a man gets sick he immediately goes either to the health centre or to the holy water site*. *But women*, *who are not able to do so*, *stay at home perceiving the problem to be located with their father’s and the mother’s spirit*. *Those who beg the spirits do not leave the house*. *That is why we*, *the women*, *suffer more*. *It is because we go to the health centre only when we have a more serious condition’*. Advice given by friends or relatives also impacted on the women’s health care decisions: ‘*Half of the people used to say that the cause of it is “qole” [spirits]*, *while the others said that the only solution is the clinic*. *My husband refused the idea of the “qole” since he is a priest*. *Instead he wanted me to go to the clinic*. *I was in dilemma and said “yes” to both “qole” and clinic*. *I was so confused’* (32-year-old married woman, treated for prolapse stage III).

The community health workers, who had the responsibility to follow up with the women for their scheduled appointment at the hospital through the campaign, observed a clear tendency among the women: *‘After knowing their appointment [for surgical treatment at the hospital] most of them went to holy water sites until their appointment time’*, one health extension worker explained. Furthermore, the women in the focus group discussions explained that the use of holy water and treatment offered at the health facilities were closely interlinked: *‘People use both*. *Those who use the “tsebel” can later get treated going to doctor*. *God will follow them there*. *People who were on medication [from the health facility] can also get treatment by the “tsebel”*. *Thus*, *the two go together*. *We consider them as brother and sister’*. The health personnel at the hospital also frequently observed that the women would go to holy water sites to bring bottles of the blessed water to the ward when admitted for surgical treatment. The health care workers stated that they, along with most people in the area, believed in the healing power of *‘tsebel’*: *‘I don’t say anything against it because it is a belief*, *and they believe more in God*. *Almost all Ethiopians are serious in their beliefs*. *I tell them that “first there is another treatment [surgery]*, *and then afterwards you can go to a holy water site”‘* (Registered nurse, hospital).

Among the women who had sought help from local healers some experienced being referred elsewhere, most often to the *‘tsebel’* sources: ‘*My brothers took me to an ‘awaki’ when my condition got severe*. *She [the ‘awaki’] told me to go to two different ‘tsebel’ sites to get cured’* (42-year-old married woman, prolapse stage III). The women in the focus group discussions expressed similar experiences: ‘*When you go to local healers the healer can tell you to go to the doctors [health facilities]*. *They [local healers] tell us if it [the ailment] is beyond their capacity’*. The local healers that we talked to appeared clear about their limitations and what to do if they failed to treat a condition: *‘If people are not healed after my treatment I suggest them not to return back*. *Instead I tell them to find a doctor*. *I don’t give them useless words*. *I sent some of them [women with prolapse] to treatment at a clinic*, *although they didn’t get a cure there*. *(…) I don’t provide anything to drink; instead I suggested them to go to a ‘tsebel’ site’*, an elderly ‘*awaki*’ man explained.

### Changing scenarios?

The health care seeking by the women suffering from prolapse in the study area was presented as a landscape characterised by changing conditions. One of the women with prolapse explained to us how spiritual healers had reduced in number in her community: ‘*I went to those who believe in a spirit different from God once or twice*. *They have vanished these days*, *but they used to help people in past times’* (70-year-old married woman, not yet diagnosed). The health workers also had similar perceptions: *‘Before there were cults*, *they called it their mother’s and father’s spirit [‘qole’]*. *Even without going to the witch houses they used to beg the spirit’s at their home*. *But now there is no such type of practice*. *At least they don’t tell me about it*, *or maybe they hide it from me’*, one health extension worker stated. Indeed, according to our participants in the focus groups, local spiritual beliefs were not easy to speak openly about and were shrouded by secrecy: *‘Of course we go there [to the local healers]*, *but we don’t tell people about it*. *Many people keep it secret*. *The priests do not allow it*. *Therefore those who go to traditional healers don’t talk about it*. *People say that they have been to Holy Water or to health centres instead’*, one participant explained.

The newly introduced community mobilisation programme for prolapse treatment had in a short time dramatically increased the knowledge and awareness of the available surgical treatment among our informants. *‘After the campaign started*, *women suffering from prolapse want to go and get treated [at the hospital]*. *When I compare this to previous times when women didn’t know that the condition had a treatment they linked it to “qole” [spirits]*. *Now they have understood that going to ‘tsebel’ or other places don’t help them’*, a health extension worker explained. As a result of the newly gained awareness in the area, previous perceptions of surgery were also said to be slowly transforming: ‘*I accepted it [surgical treatment] because the condition was so painful*. *I couldn’t wait for my turn*. *I wasn’t scared; I just wanted to be treated*’, a 38-year-old widowed woman stated. The increased openness that the new opportunity created moreover seemed to generate new hopes: *‘Previously everyone was keeping it to themselves because there wasn’t any treatment*, *but now things are getting better and many people are getting hopes of being treated’* (39-year-old divorced woman, treated for prolapse stage II).

## Discussion

Through the stories of Hanna, Aberash and Rahel we have attempted to illustrate how women’s health care decisions have been strongly influenced by poverty and a lack of knowledge about possible ailments, by religiously and spiritually grounded beliefs, by shame and embarrassment related to the condition and by limited decision-making power in the household. Experiences of no improvement after years of repeated attempts made many lose faith in the diverse available options of care and cure found in their communities, or indeed in any available solution. Due to the provision of free surgical treatment at the hospital, the awareness of prolapse as a common maternal morbidity was increasing rapidly among the women in the area during the period of this research, seemingly quite dramatically altering women’s health care preferences and choices.

### Manoeuvring between available health care options

The material demonstrates examples of how the women manoeuvred between health facilities, local healers and holy water in search of healing and a cure for their prolapse condition. *‘Tsebel’*, or holy water, proved to be highly endorsed and socially acceptable and was found to be acceptable in combination with treatment provided both at the health facilities and at the local healers. Spiritual practices had, in contrast to earlier documented findings in the area [26], in recent years taken on a more private character and were surrounded by varying degrees of secrecy. Some health workers even mentioned that these options did not exist any longer. We did however find that ceremonies to appease the spirits continue to take place albeit are not talked openly about. The ceremonies take place at the local healers’ homes or at the women’s own homes with only a few select people invited. Although some women claimed that local healers’ practises oppose their Orthodox Christian beliefs, the local healers on their side referred many of their clients to holy water.

In a study on cervical cancer in Ethiopia, the majority of the affected women did not perceive ‘modern’ medicine as the preferred mode of treatment as they believed the disease to be caused by supernatural powers. Various ‘traditional’ remedies as well as holy water were thus exhausted before seeking help at health facilities [27]. In studies from Nepal, women with prolapse had used various modes of self-care or ‘traditional’ remedies or consulted ‘traditional’ healers prior to visiting the health facilities [28, 29]. Similarly, many of our informants sought help through local or religious remedies before reaching the health facilities.

### The role of socio-cultural embedded attitudes and beliefs

The role of culture in creating and maintaining multiple meanings of illness and suffering has been extensively documented in medical anthropology [30]. We know from previous research that women with prolapse from this area have a range of ways of conceptualising and explaining the cause of their prolapse, from childbirth and labour to food scarcity, heavy workloads, God’s will or linked to spirits such as the ‘evil eye’ [13]. Among Orthodox Christians, good health is commonly regarded as a ‘gift of God’ [19], and throughout our study we witnessed how the religious holy water served as an integral and natural part of peoples’ lives unrelated to peoples’ wealth or status. From other parts of the Amhara region, there are similar illustrations of how health personnel incorporate religious convictions into their biomedical understanding [21], which reflects the deeply rooted belief in the healing attributes of holy water in the society.

Also deeply rooted in the study area is the common belief that disease is caused by supernatural forces [19]. Only certain individuals are believed to have the possibility of directly accessing the ‘*zar*’ spirits, such as the ‘*awaki*’ or ‘*tenquay*’ healers [20, 31]. According to Messing [26], who studied the common phenomenon of ‘*zar cults’* in the Amhara region some 60 years ago, people considered every human as potentially vulnerable to be possessed by a *‘zar’* spirit which could cause illness. Also, the belief that the ‘evil eye’ can cause sickness or death is strongly embedded in the culture in the region [32]. Although this is a context constantly in transformation, our material suggests that such spiritual beliefs still play an important role in the study area. On a daily basis, people in the communities strove to uphold a good relation to and protect themselves from potential evil spirits through the carrying out of various deeds or use of symbols such as the ‘*kitab*’ (protective amulet). When an illness struck, many turned to a local spiritual healer or holy water for help and assistance to restore their relationship with or expel the angry or evil ‘spirits’.

### Structural barriers to reaching health care facilities

From a critical medical anthropology perspective the susceptibility to disease is strongly linked as to social factors such as poverty and economic insecurity, malnutrition, housing and political powerlessness [33, 34]. Social inequality, class, gender, ethnicity or poverty may serve as immense barriers to achieving wellbeing and accessing quality biomedical care [35].

Kassaye et al. [19] argue that one of the biggest challenges in Ethiopia lies in how to narrow the gap between existing biomedical services and the large rural population with limited access to these services. They claim that people to a large extent rely on ‘traditional’ medicine in rural areas of Ethiopia due to its relatively low cost and due to the difficult access to health facilities. Although the consultancies by local healers or the conduction of repeated spiritual ceremonies were by some considered costly, the use of local healers and by holy water sites were overall more easily accessible for the women in this area.

As reported from studies on prolapse in other resource-constrained settings [7, 10, 29, 36], the majority of the women in the present rural context were illiterate. They were also economically dependent on their husbands, had long distances to the nearest health centre which offered limited relevant services and moreover had difficulties leaving their home due to their responsibility for children and household chores. It is thus clear that the structural barriers to reaching appropriate health services were substantial and severely limited the women’s awareness of the condition and the possibility to reach the health facilities. The women’s power to make decisions was moreover hampered through very early marriage, which for many had implications for the possibility to go to school and for early pregnancies, which again are associated with high risk of obstetric complications [37]. Women’s autonomy and freedom to make decisions are moreover largely inhibited by patriarchy and social norms in the Ethiopian rural context, and this consequently has a negative impact on health care seeking among women [38]. It was clear that the women’s delay in health care seeking was strongly embedded in the severe structural prohibitions that characterised their life contexts. Hence, the women’s extended years of suffering with prolapse may be understood as a phenomenon influenced by the ‘*socially constituted categories of meaning and the political-economic forces that shape daily life*’–what Singer refers to as a ‘social product’ [34].

### Factors enabling women to access treatment

The issue of patient costs as a barrier to accessing health care for the worst off has been widely recognised, and more services are today being provided at reduced or no cost [39], such as through the mentioned exemption schemes and waived fees in Ethiopia. However, ‘free’ services are often still perceived as costly by Ethiopian village standards as they do not necessarily cover all the indirect costs that a hospital stay entails, such as transport, accommodation and food for one’s family while hospitalised.

In spite of the evidence of immense barriers to adequate health services, it has been reported that people may be pragmatic and willing to change or modify their preferences in health care if they perceive the change as a realistic possibility and they see that the biomedical treatment offered is more effective [39]. Hence, when people experience that the economic costs are within their capacity and the social costs acceptable, there is evidence that people may be willing to embrace new services. In our study context, where a new initiative provided women with knowledge and with practical arrangements including transport as well as the financial coverage of a treatment, a quite dramatic transition seemed to take place. The women in the present study found acceptable avenues to combine the holy water with treatment at the hospital and to bracket their spiritual beliefs and their fear of surgery and willingly accepted the surgical treatment provided at the hospital.

The present study indeed seems to serve as an interesting example of how removing structural barriers may facilitate modification in customary health seeking conduct and access to effective healing. Particularly interesting is moreover the manner in which access to successful healing of shameful maternal health conditions was shown, through another sub-study not yet published focusing on recovery and reintegration among the same patient group, to be followed by a rapid decrease in the shame and secrecy surrounding the condition. One may expect that with time a retained free-of-charge service may increasingly lead women to disclose their conditions in order to receive or at least seek help from the health facilities at a far earlier stage, alleviating them from years of suffering and shame.

### Strength and limitations of the study

The first author’s socio-cultural and language limitations are likely to have affected the study results. In this context it may also be mentioned that being an outsider can at times be an advantage as one is perceived to be located beyond the locally embedded normative discourse and thus less dangerous to share experiences with. The current study was moreover based upon fieldwork carried out over an extended period of time and thus allowed for follow up with many participants. Interviews with multiple categories of informants coupled with focus group discussions and participant observations ensured triangulation, which enhanced the trustworthiness of the data. The recruitment of women suffering from prolapse was limited to rural and semi-urban women enrolled in the ongoing campaign. Although one would expect urban women´s experiences of health care seeking to differ from those of rural, we do not expect substantial difference among the women experiencing prolapse and who are not enrolled in the campaign within the rural and semi-urban communities due to the shared structural and cultural barriers of health care seeking within the public health system. As most of the interviews were carried out at the hospital, this might have limited the women’s willingness to talk openly about their potential previous health care seeking experiences, especially those related to spiritual healing options. However, the follow up interviews carried out in their homes in the months following their hospital admission were found to have to some degree ameliorated this limitation.

## Conclusions

This study highlights some of the dynamics taking place between the public health system and alternative healing options in a resource-constrained setting where women suffering from a common and treatable maternal morbidity search for cure and healing. Severe conditions of poverty and a lack of knowledge about the condition they are suffering from as well as about available treatment serve as the main barriers to reaching the health facilities. Our results furthermore illustrate how the provision of free quality services may dramatically alter both health- and illness-related perceptions and conduct in an extremely vulnerable population. The present study speaks to the increasing calls for addressing the large scale of maternal morbidities that severely impacts the lives of women living in resource-constrained settings.
